# Four and a Half LIM Protein 1C (FHL1C): A Binding Partner for Voltage-Gated Potassium Channel K_v1.5_

**DOI:** 10.1371/journal.pone.0026524

**Published:** 2011-10-28

**Authors:** Ivana Poparic, Wolfgang Schreibmayer, Benedikt Schoser, Gernot Desoye, Astrid Gorischek, Heidi Miedl, Sonja Hochmeister, Josepha Binder, Stefan Quasthoff, Klaus Wagner, Christian Windpassinger, Ernst Malle

**Affiliations:** 1 Institute of Human Genetics, Institute of Molecular Biology and Biochemistry, Medical University of Graz, Graz, Austria; 2 Institute of Biophysics, Institute of Molecular Biology and Biochemistry, Medical University of Graz, Graz, Austria; 3 Department of Neurology, Friedrich Baur Institute, Ludwig Maximilians University of Munich, Munich, Germany; 4 Department of Obstetrics and Gynaecology, Institute of Molecular Biology and Biochemistry, Medical University of Graz, Graz, Austria; 5 Department of Neurology, Institute of Molecular Biology and Biochemistry, Medical University of Graz, Graz, Austria; 6 Department of Cardiology, Institute of Molecular Biology and Biochemistry, Medical University of Graz, Graz, Austria; 7 Center for Molecular Medicine, Institute of Molecular Biology and Biochemistry, Medical University of Graz, Graz, Austria; University of South Florida College of Medicine, United States of America

## Abstract

Four-and-a-half LIM domain protein 1 isoform A (FHL1A) is predominantly expressed in skeletal and cardiac muscle. Mutations in the *FHL1* gene are causative for several types of hereditary myopathies including X-linked myopathy with postural muscle atrophy (XMPMA). We here studied myoblasts from XMPMA patients. We found that functional FHL1A protein is completely absent in patient myoblasts. In parallel, expression of FHL1C is either unaffected or increased. Furthermore, a decreased proliferation rate of XMPMA myoblasts compared to controls was observed but an increased number of XMPMA myoblasts was found in the G_0_/G_1_ phase. Furthermore, low expression of K_v1.5_, a voltage-gated potassium channel known to alter myoblast proliferation during the G_1_ phase and to control repolarization of action potential, was detected. In order to substantiate a possible relation between K_v1.5_ and FHL1C, a pull-down assay was performed. A physical and direct interaction of both proteins was observed *in vitro*. In addition, confocal microscopy revealed substantial colocalization of FHL1C and K_v1.5_ within atrial cells, supporting a possible interaction between both proteins *in vivo*. Two-electrode voltage clamp experiments demonstrated that coexpression of K_v1.5_ with FHL1C in *Xenopus laevis* oocytes markedly reduced K^+^ currents when compared to oocytes expressing K_v1.5_ only. We here present the first evidence on a biological relevance of FHL1C.

## Introduction

LIM, an acronym of three homeodomain-containing transcription factors (Lin-11, Isl-1, and Mec-3), contains a 50–60 amino acid stretch and a highly conserved, double cysteine-rich Zink-finger domain. LIM domain-containing proteins play important roles in various cellular processes, such as cytoskeleton organization, signal transduction, gene expression and cell differentiation [1], [2]. Four and a half LIM protein 1 (FHL1) is a main representative of the LIM-only protein family that is characterized by a half LIM domain (located in the N-terminal portion) and four additional complete LIM domains stretching towards the C-terminal portion. FHL1 is suggested to play a role in sarcomere synthesis and assembly [3]. Overexpression of FHL1 in mouse skeletal muscle promotes myocardial fusion and hypertrophy, observations with potential implications for human myopathy [4]. In humans, FHL1 is predominantly expressed in skeletal muscle and heart, but also in other tissues e.g. brain, placenta, lung, liver and kidney, at a lower abundance.

**Figure 1:**
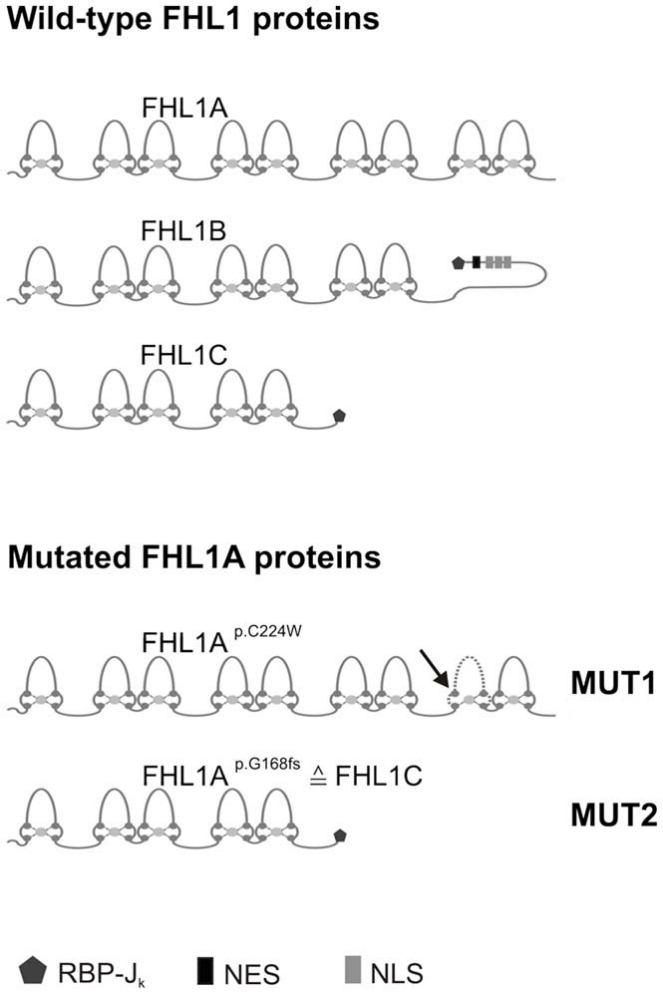
Wild-type FHL1 protein isoforms and mutated FHL1A proteins. *Top*: FHL1A, FHL1B and FHL1C protein structures (resulting from alternative splicing of the wild-type FHL1 gene), including the LIM domain architecture and functional domains (*RBP-Jk*, recombination signal-binding protein 1 for J-kappa; *NLS*, nuclear localization signals; *NES*, nuclear export sequence) are shown for the corresponding variants. *Bottom*: Protein structures resulting from alternative splicing of the two different mutant FHL1 genes. An arrow in the fourth LIM domain of mutated FHL1A at position 224 (termed FHL1A^p.C224W^) indicates the position of the amino acid exchange. The dotted line suggests alterations in the LIM-like architecture (termed MUT1 here). FHL1A^p.G168fs^ is identical to FHL1C protein (termed MUT2 here) (G = glycine, fs = frame shift).

In addition to the prevalently expressed FHL1A protein (280 amino acids) two minor splice variants, originally identified from murine studies, exist. FHL1B (323 amino acids, the homolog of murine KyoT3 and specifically expressed in brain [5]) comprises the N-terminal three and a half LIM domains and a unique C-terminus containing three nuclear localization signals, a nuclear export sequence, and a C-terminal binding site for RBP-Jk. FHL1C (194 amino acids, the homolog of murine KyoT2) contains the N-terminal two and a half LIM domains and the C-terminal binding site for RBP-Jk (Figure 1).

Several mutations in the human *FHL1* gene are associated with severe or less severe types of hereditary muscular dystrophies and myopathies [2], [6]–[11]. The majority of these FHL1 mutations are located in the second LIM domain of FHL1A. A specific mutation in the fourth LIM domain in the *FHL1A* gene as occurring is associated with X-linked myopathy with postural muscle atrophy (XMPMA, MIM ID #300696). This disease is characterized by atrophy of postural muscles with rigid spine syndrome, pseudo athleticism or hypertrophy and hypertrophic cardiomyopathy [6].

The present study aimed at investigating myoblast proliferation from two XMPMA patients with different mutations in *FHL1A*. The classical missense mutation c.672C>G (on protein level p.C224W) leads to disruption of the fourth LIM domain in FHL1A and subsequent degradation of the protein due to the loss of one cysteine-rich Zink-finger domain; in parallel expression of endogenous FHL1C remains unaffected [6]. The second, a splice site mutation c.688+1G>A (on protein level p.G168fs), results in the deletion of two C-terminal LIM domains in FHL1A; the resulting truncated FHL1-like protein is identical to FHL1C (Figure 1); as a consequence, concentrations of FHL1C (the sum of endogenous and truncated FHL1-like protein) should increase [9]. FHL1C is specifically expressed in testis, skeletal muscle, and heart at a relatively low level compared to FHL1A [12]; however, to date FHL1C is still considered a protein of unknown function.

Recent evidence suggested that the voltage-gated potassium channel K_v1.5_ could act as a binding partner for LIM-domain containing proteins [13]. In myoblasts, cell cycle-dependent expression of K_v1.5_ has been reported [14]. K_v1.5_ plays a role in myocardial repolarisation and may regulate skeletal muscle proliferation [14], [15]. Furthermore, K_v1.5_ forms the major basis of an atrial-specific ultra-rapid delayed rectifier potassium ion current *I*_Kur_ [16] and most importantly, a missense mutation in K_v1.5_ can result in atrial fibrillation [17]. Therefore, the second aim of this study was to clarify whether K_v1.5_ is expressed in XMPMA myoblasts and whether this K^+^ channel could act as a specific binding partner for FHL1C. We further investigated whether FHL1C and K_v1.5_ colocalize in cellular compartments. Using a pull-down assay we then tried to reveal a direct interaction between both proteins. Finally, electrophysiological studies were performed, aiming to test whether FHL1C functionally interacts with K_v1.5_.

## Materials and Methods

### Cell culture

(i) Human myoblast cultures were established from muscle biopsies of two male unaffected controls (termed WT1 and WT2) and two male XMPMA patients with either the missense mutation p.C224W (termed MUT1) or the splice mutation p.G168fs (termed MUT2) (Figure 1). Clinical characteristics and histological examinations in addition to molecular genetics of MUT1 and MUT2 patients are described in detail in reference [9]. The ethic board of the Ludwig-Maximilians University, Munich, Germany, gave ethical approval. All patients gave their written informed consent to scientific analyses of their blood samples and myoblasts.

Tissue sources for myoblast isolation were tibialial anterior muscle biopsy (WT1 and MUT1) and a biceps brachii muscle biopsy (WT2 and MUT2). Myoblasts were grown in skeletal muscle cell growth medium (PromoCell GmbH, Heidelberg, Germany) including supplement mix containing 10% (v/v) FCS (Invitrogen, Lofer, Austria), 1.5% (v/v) 100× Glutamax, 50 µl/ml gentamycin. Myoblasts isolated from controls and XMPMA patients consisted of a homogenous cell population and were confirmed to be of satellite origin (using a specific antibody raised human neuronal cell adhesion molecule, clone 123C3, Monosan, Uden, The Netherlands).

(ii) HL-1, established from the AT-1 mouse atrial cardiomyocyte cell line, was used for cotransfection experiments [18]. Cells were grown in Claycomb medium (Fluka, Sigma Aldrich, Munich, Germany) including 10% (v/v) FCS at 37°C (5% CO_2_, 95% air, relative humidity of 95%).

### Myoblast proliferation assay

Myoblasts (2×10^5^ cells, 40% confluence) were seeded in six-well plates at a density of 2.7×10^4^/cm^2^ cultured up to 96 h in skeletal muscle cell growth medium (including a supplement mix containing 10% [v/v] FCS, 1.5% [v/v] 100× Glutamax, 50 µl/ml gentamycin). Cells were harvested after 24, 48, 72 and 96 h using 0.25% (v/v) trypsin. Cell proliferation was determined by measuring the number of viable cells at the indicated time periods using a cell counter and analyser system CASY®Model TT (Roche Innovatis AG, Bielefeld, Germany) as described [19].

### Cell cycle analysis by flow cytometry

Myoblasts were seeded in six-well plates at a density of 2.7×10^4^ cells/cm^2^ and cultured for 48 h, collected in PBS and centrifuged 5 min at 200 *g*. Cells were resuspended in 0.5 ml PBS and fixed in ice-cold 70% ethanol overnight. Thereafter, 0.25% (w/v) pepsin was added followed by CyStain DNA/protein staining solution. Samples were kept for 30 min at 4°C in the dark. Flow cytometric measurements were carried out using CyFlow® flow cytometer (Partec) according to the manufacturer's suggestions. For data analysis MultiCycleAV DNA cell cycle analysis software (Phoenix, San Diego, CA, USA) was used.

### RNA isolation and reverse transcription PCR

Myoblasts (2×10^5^ cells) were seeded in six-well plates, cultured for 48 h and harvested by treatment with 0.25% (v/v) trypsin. TRI Reagent (Invitrogen) was used for total RNA isolation according to the manufacturer's suggestions.

Final RNA concentrations were measured by Nanodrop technique (Thermo Scientific). Reverse transcription was performed from two µg of total RNA with random hexamer primers and SuperScript III Reverse Transcriptase (RT) (Invitrogen) [20], [21]. PCR primers (Table S1) were used to amplify the corresponding PCR products for FHL1 and K_v1.5_. To ensure equal RNA loading, RT-PCR for human hypoxanthine phosphoribosyltransferase was performed for each experiment. Two control reactions (RNA template without primers and a water template only) were included in the absence of cDNA.

### Protein isolation and Western blotting

Myoblasts were cultured and trypsinized as described above. The cells were washed with HBSS buffer, mixed with lysis buffer (0.1 M Tris pH 7.4, 10% [v/v] SDS, 0.1 M Na_3_VO_4_, 0.5 M NaF, 0.1 M sodium pyrophosphate buffer) and heated at 95°C for 10 min. Total cell lysate was frozen and stored at −20°C until use. Protein concentrations were measured with the Bradford Protein Assay Kit (Bio-Rad, Vienna, Austria). Aliquots of proteins were supplemented with NuPage® LDS sample buffer and NuPage® sample reducing agent (5%, v/v). After boiling for 5 min at 95°C, protein samples were subjected to electrophoresis at 4–12% NuPage® gradient SDS-PAGE gels [22] and transferred (90 min, 200 mA) to nitrocellulose membranes (0.45 µm, Schleicher and Schuell, Dassel, Germany, [23]). The membranes were blocked (30 min) with TBST (10 mM Tris-HCl [pH 7.4], 140 mM NaCl, 0.1% [v/v] Tween-20) containing 3% (w/v) non-fat dry milk followed by incubation overnight at 4°C with polyclonal rabbit anti-human FHL1 antibody (1∶1000, cross-reacting with the fourth LIM domain [coded for by exon 4–6] of FHL1A, AVIVA Systems Biology, San Diego, CA, USA) or polyclonal goat anti-human K_v1.5_ (1∶200, Santa Cruz Biotechnology, Inc., Santa Cruz, CA, USA) as primary antibodies (diluted in 3% (w/v) BSA). After washing, the membranes were incubated (2 h, 25°C) with swine anti-rabbit IgG (1∶750) or donkey anti-goat IgG (1∶2000) as secondary antibodies (diluted in TBST containing 3% [w/v] non-fat dry milk). Immunoreactive bands were visualised with Immobilon Western Chemiluminescent HRP substrate (Millipore, Billerica, MA, USA). For normalisation, membranes were stripped with Restore™ Western Blot Stripping buffer (Pierce) and reprobed with anti-β-human actin antibody (1∶1000, in 3% [w/v] BSA, Santa Cruz) and goat anti-mouse IgG (1∶2500, 1 h, 25°C, Rockland, Gilbertsville, PA, USA) as secondary antibodies.

### HL-1 cells transfection and confocal microscopy

FHL1C-p^eYFP^-N1 (plasmid encoding for human FHL1C with the enhanced yellow fluorescence protein fused to its C-terminus) and K_v1.5_-p^eYFP^-N1 clones were raised using PCR technique and suitable primers (Table S2; see below). The p^eCFP^-C1+human K_v1.5_ construct (plasmid encoding for human K_v1.5_ with the enhanced cyan fluorescence protein fused to its N-terminus) was kindly provided by Dr. Antonio Felipe (Barcelona, Spain). Plasmids encoding for eCFP-labelled ß-subunit of the signal recognition particle receptor Srß (Srß^eCFP^) and the eCFP-labelled glycosylphosphatidylinositol anchor domain (GPI^eCFP^) were kindly provided by Dr. Koret Hirschberg (Tel Aviv, Israel) and prepared as described [24], [25]. Srß^eCFP^ was used as an endoplasmic reticulum marker while GPI^eCFP^ was used to label lipid raft domains within the plasma membrane.

Transfection of HL-1 cells with the plasmids mentioned above was performed with Lipofectamine according to the manufacturer's suggestions. Cells were screened for signal 24 to 48 h after transfection using confocal imaging using a Leica inverted microscope with a laser-scanning module attached (DMIRE2 and TCS SL2; Leica Microsystems, Heidelberg, Germany). Using the 63×water immersion objective (NA: 1.20), confocal sections (1024×1024 pixels) were obtained at 12-bit resolution. Filter settings were: *eYFP*: excitation (514 nm); excitation beam splitter: DD 458/514; emission (540–570 nm). *eCFP*: excitation (458 nm); excitation beam splitter: DD 458/514; emission (477–500 nm). Interference between eCFP and eYFP channels was negligible. Analysis of confocal images was done using the ImageJ software (ImageJ 1.42 h by Wayne Rasband, NIH, and Bethesda, MD) supported with deconvolution plug-in (Bob Dougherty; http://www.optinav.com/imagej.html).

### Pull-Down assay

A DNA fragment encoding amino acid residues 518 to 613 (numbering according to human K_v1.5_, NP_002225.2) was synthesized by the PCR using a cDNA clone encoding full length K_v1.5_ (kindly provided by Dr. Joachim Ehrlich, Frankfurt am Main, Germany) as a template and cloned into the vector pGEX-4T-1 using suitable primers (Table S2). The recombinant protein was heterologously expressed in *E.coli* and purified as described [26]. Four hundred fifty ng cRNA encoding FHL1C (or Gβ_1_ and Gγ_2_, respectively) were translated and radiolabelled *in vitro* using 9.4 µl rabbit reticulocyte lysate (Promega, Mannheim, Germany) and 0.95 µl ^35^S-methionine (Amersham GE Healthcare) by incubation at 37°C for 2 h. Purified GST-K_v1.5_ fusion protein (10–20 µg) was used as bait and incubated with 10 µl of lysate containing ^35^S-labeled FHL1C in 300 µl buffer (25 mM HEPES-Na, 5 mM MgCl_2_, 5 mM EGTA, 0.05% Tween-20 [v/v, pH 7.0]), for 2 h at 25°C, with gentle rocking. GST protein alone was used as a negative control (i.e. no binding to FHL1C was expected). G-protein activated inwardly rectifying potassium channel (GIRK1) C-terminus (G1-CT) was used both as a positive control (in combination with ^35^S-labeled G-protein β subunits (Gβγ)) as well as a negative control (in combination with ^35^S-labeled FHL1C lysate). Control proteins were prepared as described [26]. Then, 30 µl of previously washed Glutathione Sepharose beads were added, the mixture was incubated (30 min, 25°C) and then washed three times in 1 ml of the same buffer. After washing, GST-fusion proteins were eluted with 30 µl of 15 mmol/l reduced glutathione in elution buffer (120 mM NaCl, 100 mM Tris, 0.05% Tween-20 [v/v, pH 8]) and subjected to 12% linear SDS-PAGE. Radioactivity was quantified by autoradiography of the dried gels, using a PhosphorImager Storm™ (GE Healthcare, Vienna, Austria). The amount of radioactivity was normalized to the amount of protein of a given band, as measured by staining of bands with Coomassie Brilliant Blue ( = relative specific radioactivity).

### DNA constructs

For protein expression in *Xenopus* oocytes, FHL1C and K_v1.5_ inserts were synthesized by PCR using suitable primers (Table S2) and cloned into the pBSmxt oocyte expression vector. A full-length human cDNA library (human fetal brain marathon cDNA) and K_v1.5_-pcDNA3.1 (kindly provided by Joachim Ehrlich, Frankfurt, Germany) were used as PCR templates. The resulting pBSmxt constructs were linearized, cRNA was synthesized as described [27], aliquots were shock frozen in liquid nitrogen and stored at −70°C until use. Integrity of all DNA constructs was verified by DNA-sequencing (3130xl DNA Analyzer, Applied Biosystems, Vienna, Austria). DNA was prepared by Qiagen midiprep kit and concentration and purity was checked with Nanodrop (Thermo Scientific, Waltham, MA, USA).

### *Xenopus* oocytes expression and electrophysiology

*Xenopus* oocytes, prepared as described [28], were injected with 50 nl of the following RNAs (either alone or in combination): K_v1.5_ (2 ng RNA/nl), FHL1C (50 or 500 ng RNA/nl). Oocytes were incubated in ND96 solution (96 mM NaCl, 2 mM KCl, 1 mM CaCl_2_, 1 mM MgCl_2_, 5 mM HEPES/NaOH [pH 7.6]) supplemented with gentamycin (50 µg/ml) and sodium pyruvate (2.5 mM) at 19°C. Three to seven days after injection of oocytes, whole cell currents were recorded using agarose cushion electrodes [29]. K_v1.5_ channels were characterized by the following voltage jump protocols: voltage-dependent activation was assessed by keeping the oocytes at a resting potential of −80 mV and K^+^ currents were elicited by consecutive voltage jumps repeated every 7 sec (from −35 mV to +45 mV in 5 mV increments). Inactivation of K_v1.5_ was quantified by the ratio of the magnitude of K^+^ current 1.8 sec after a suprathreshold pulse to +15 mV divided by the peak current at this potential. Current traces were sampled at 300 msec sampling rate and low pass filtered at 500 kHz using a 4-pole Bessel filter. Parameters were normalized to the ones obtained from control oocytes (injected with cRNA encoding K_v1.5_ alone) for a given experimental day and batch of oocytes. Data acquisition and analysis was performed using the digidata 1322A interface and the pClamp 9.2 software (both Molecular Devices, Sunnyvale, CA, USA).

### Statistics

Each experiment was performed at least three times. Data of representative experiments are expressed as mean ± SD or ± SEM. Experimental parameters were analyzed between groups using Student's *t*-test (Sigma plot for Windows, v11, Systat Software). Means were checked for statistically significant differences (* p<0.05, ** p<0.01, and *** p<0.001). Sequence analysis and alignments were performed using Chromas Lite (v2.01, Technelysium Pty Ltd).

## Results

### Myoblast proliferation and cell cycle distribution

**Figure 2:**
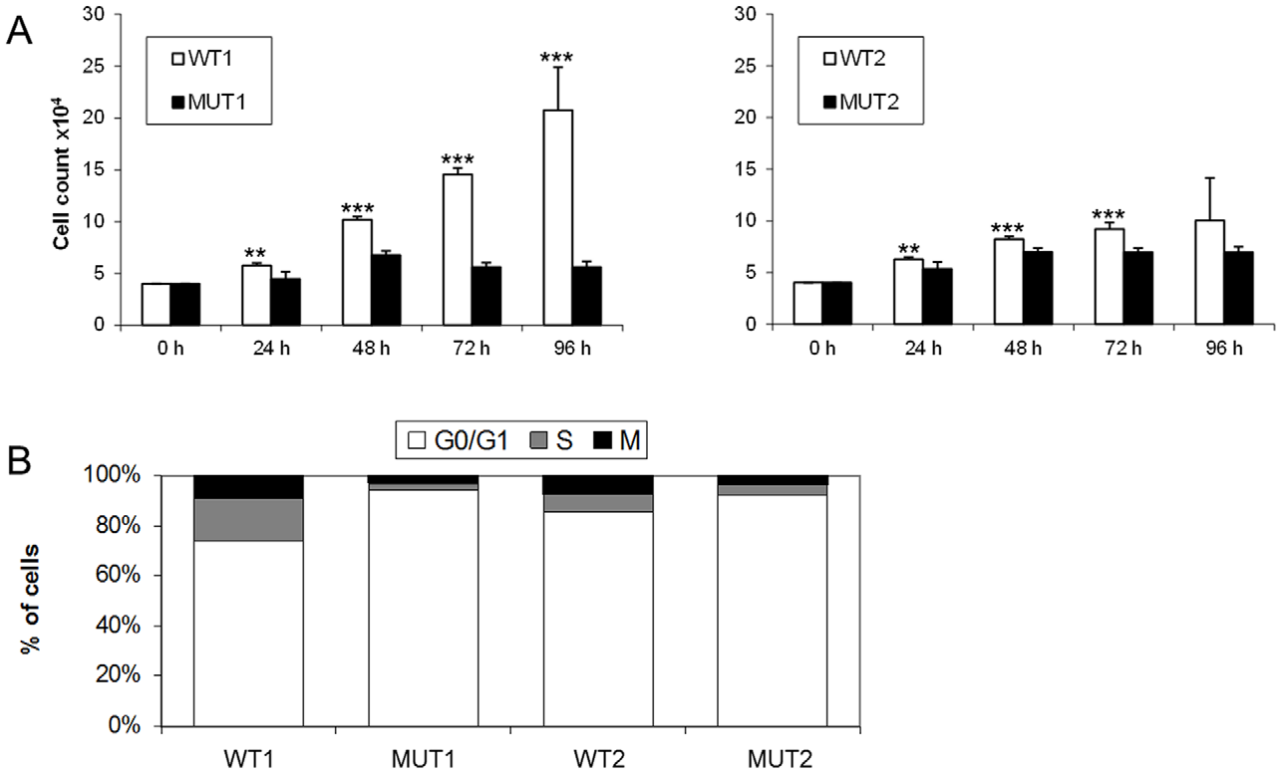
Proliferation rate of human myoblasts. Myoblasts from controls (WT1, WT2) and patients (MUT1, MUT2) were cultured for the indicated times (A) or for 48 h (B). Myoblast samples WT1 and MUT1 originated from tibialial anterior muscle biopsy, samples WT2 and MUT2 from biceps brachii muscle biopsy. (A) Cell proliferation was determined by measuring the number of viable cells using Casy cell counter. Values represent mean ± SD of three experiments (six wells per one experiment). **p<0.01 and *** p<0.001. (B) Flow cytometric measurements (see Methods) were performed to estimate cell cycle, i.e. G_0_/G_1_ phase, S phase, and M phase, respectively. One representative experiment (performed in triplicate) out of three is shown.

A significant reduction in the number of viable myoblasts isolated from both XMPMA patients when compared to controls was observed in between 24 h and 72 h (Figure 2A). Although the differences between WT and mutant myoblasts were statistically significant up to 72 h, the differences between WT2 and MUT2 were less pronounced when compared to WT1 and MUT1. To detect possible cell cycle alterations, FACS analyses were performed. A significantly higher number of patient myoblasts was found in the G_0_/G_1_ phase when compared to controls (Figure 2B). Statistical evaluation revealed that the increase of patient myoblasts in the G_0_/G_1_ phase was 28% in MUT1 (p<0.001) but only 8% in MUT2 (p<0.001).

### Expression of K_v1.5_ and FHL1 in myoblasts of XMPMA patients and controls

**Figure 3:**
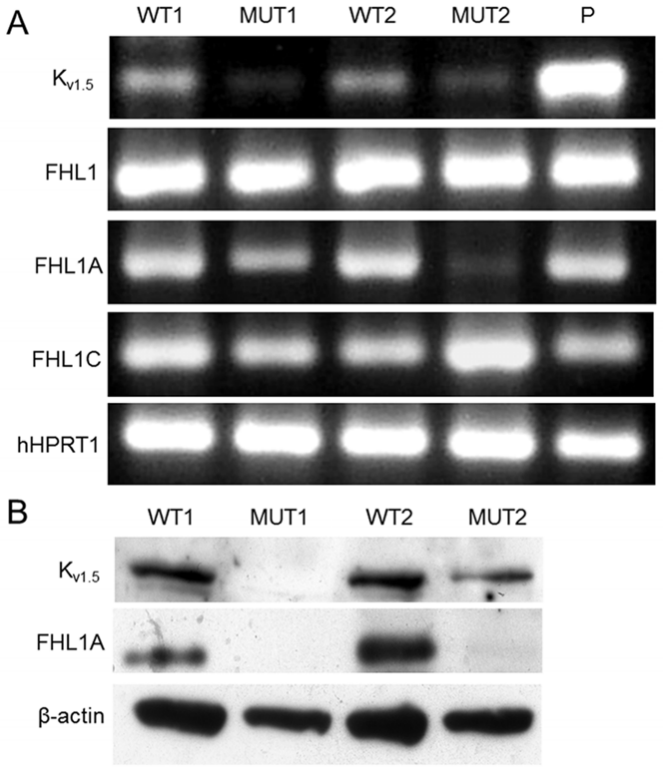
RT-PCR and Western blot for FHL1 and K_v1.5_ in human myoblasts. (A) RNA was isolated from myoblasts from controls (WT1, WT2) and XMPMA patients (MUT1, MUT2) and the corresponding K_v1.5_ and FHL1 regions were amplified by RT-PCR. Primers, spanning from exon 1 to 2 (termed FHL1) amplify the mRNA stretch coding for the common N-terminus of FHL1 in all three FHL1 isoforms (FHL1A, FHL1B and FHL1C), while primers termed FHL1A or FHL1C specifically amplify mRNAs encoding FHL1A or FHL1C, respectively. (P = positive control: human fetal brain marathon cDNA). To ensure equal gel loading, RT-PCR for human HPRT1 was performed. One representative experiment out of three is shown. See Table S1 for the primer sequences used. (B) Protein lysates from myoblasts from controls (WT1, WT2) and XMPMA patients (MUT1, MUT2) were subjected to SDS-PAGE. Proteins were transferred to nitrocellulose membranes and immunoreactive bands were detected with anti-K_v1.5_ or anti-FHL1A as primary antibodies. After stripping, the membranes were incubated with anti-β-actin antibody. (K_v1.5_ = 68 kDa; FHL1A = 32 kDa; β-actin = 45 kDa). One representative experiment out of three is shown.

Expression of K_v1.5_ on mRNA (Figure 3A) and protein level (Figure 3B) was almost absent (MUT1) or decreased (MUT2) in patient myoblasts, when compared to controls. Expression of total FHL1 mRNA transcripts (coding for all known FHL1 isoforms (FHL1A–C) is comparable between control and XMPMA myoblasts (Figure 3A). While expression of FHL1A mRNA is similar in control myoblasts (WT1 and WT2), it is remarkably decreased (MUT1) or almost absent (MUT2) in XMPMA cells (Figure 3A). In parallel, expression of mRNA encoding FHL1C was found to be low (MUT1) or high (MUT2). This observation is in line with the assumption that the production of mRNA encoding for FHL1C is unaffected in MUT1 but apparently increased in MUT2; this splice site mutation generates additional mRNA encoding a truncated FHL1-like protein that is identical to FHL1C.

Subsequently, FHL1 expression on the protein level was investigated. Due to the lack of commercially available antibodies against FHL1C only expression of FHL1A could be followed. Western blot experiments demonstrated the presence of FHL1A in myoblasts from controls while only trace amounts were present in XMPMA patient myoblasts (Figure 3). Densitometric evaluation of immunoreactive K_v1.5_ and FHL1A bands is shown in Figure S1.

### Subcellular localisation of FHL1C and K_v1.5_

Since FHL1A (a protein containing four and the half LIM domains) has recently been shown to interact with K_v1.5_ [13], we were interested whether FHL1C (lacking the two C-terminal LIM domains being present in FHL1A) could behave in a similar manner. First, we checked whether FHL1C colocalized with K_v1.5_ *in vivo*. Fluorescently labelled K_v1.5_ and FHL1C chimeras were generated by PCR technique and the corresponding constructs were expressed in atrial cells. Figure 3A shows pronounced expression and predominant nuclear localization of FHL1C^eYFP^ and, to a lesser extent, in the cytoplasm; these results partly parallel previous findings when FHL1C (tagged with the green fluorescence protein) was expressed in a hepatoma cell line [12] or embryonal fibroblasts [30]. On the other hand K_v1.5_^eCFP^ was predominantly localized within the cytoplasm with low fluorescence intensity in the nucleus.

**Figure 4:**
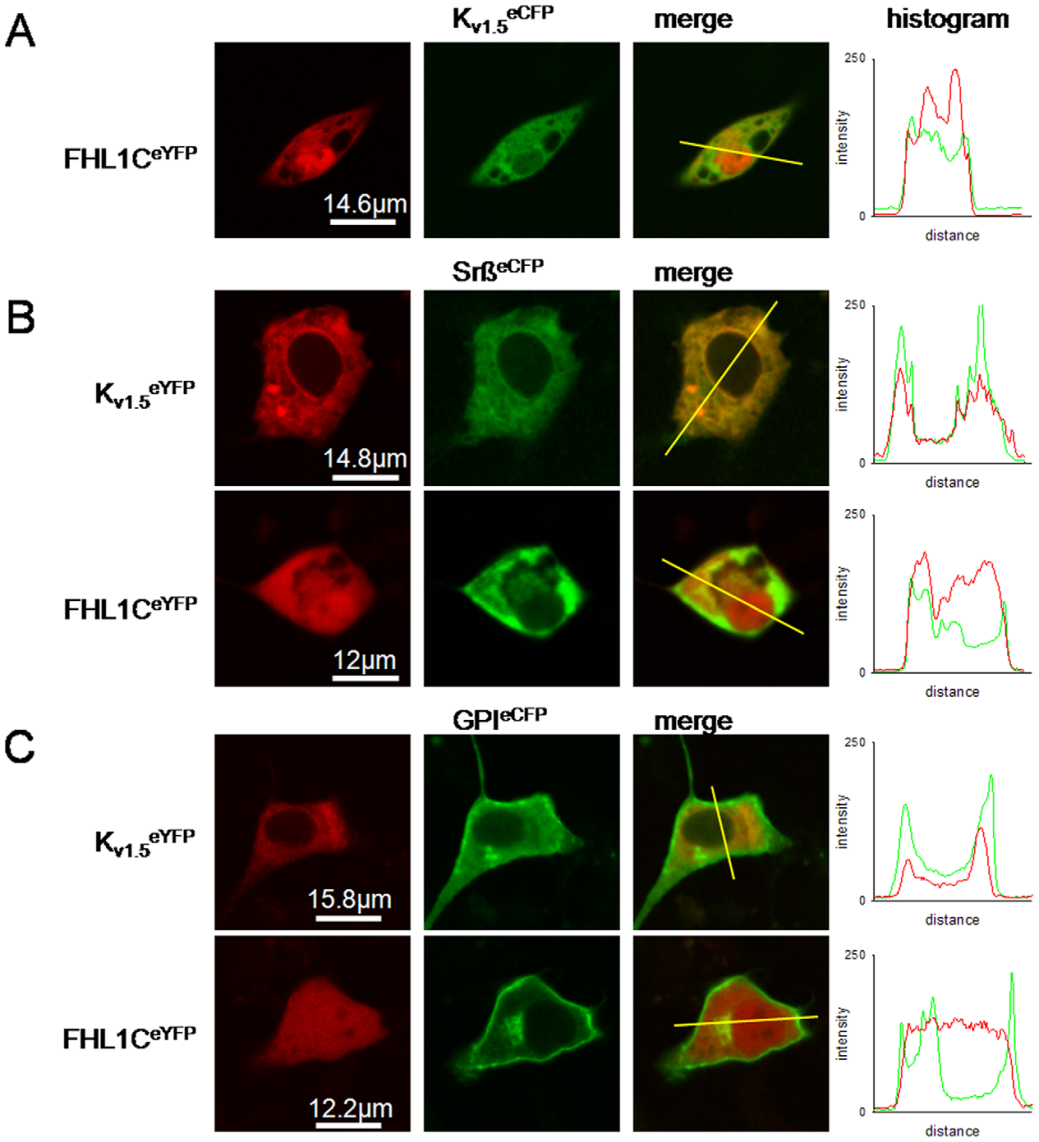
Subcellular localization of heterologous expressed chimaeric FHL1C and K_v1.5_ constructs in the atrial HL-1 cell line. Cells were cultured as described and after transfection, fluorescence of either eYFP- and eCFP-labelled proteins was detected as described in the Methods section. Each horizontal sequence of images was taken from the same cell and vertical section. For better visualization of colocalization, the eYFP and eCFP channels are shown in green and red, respectively, hence yellow in the overlay image indicates colocalization (merge). Intensity histograms are shown to quantify colocalization of both markers (right). Histogram shows the pixel-by-pixel analysis of the section indicated by the yellow line in the merged channel (from left to right). (A) Staining for FHL1C^eYFP^ and K_v1.5_^eCFP^ and colocalization. (B) Staining for K_v1.5_^eYFP^ (upper) and FHL1C^eYFP^ (lower) and colocalization with Srß^eCFP^ (a marker for the endoplasmic reticulum). (C) Staining for K_v1.5_^eYFP^ (upper) and FHL1C^eYFP^ (lower) and colocalization with GPI^eCFP^ (a marker for lipid raft domains of the plasma membrane). One representative series of images out of three independent series of experiments is shown.

The precise subcellular distribution of K_v1.5_ and FHL1C was studied by coexpression of both proteins with marker proteins that localize within specific cellular compartments. Coexpression of K_v1.5_^eYFP^ with Srß^eCFP^ revealed predominant localization of the channel in the endoplasmic reticulum (Figure 4B). Partial colocalization of K_v1.5_^eYFP^ with GPI^eCFP^ is further indicative for its additional presence in the plasma membrane including lipid rafts (Figure 4C). Distribution of FHL1C^eYFP^ showed considerable overlap with Srß^eCFP^ (Figure 4B) and partial overlap with GPI^eCFP^ (Figure 4C), indicating that substantial amounts of FHL1C localize intimately close to both subcellular compartments. Intensity histograms (Figure 4A–C) show the number of pixels in the images at each different intensity value. During our experiments we have obtained individual histograms for each signal (CFP and YFP, presented in red or green) and the combination of both is shown (Figure 4A–C). Although FHL1C and K_v1.5_ exerted quite different subcellular localization patterns as a whole, there was substantial overlap within both the plasma membrane and the endoplasmic reticulum, indicating that interaction of these proteins is likely within a living cell.

### Physical interaction between FHL1C and K_v1.5_

**Figure 5:**
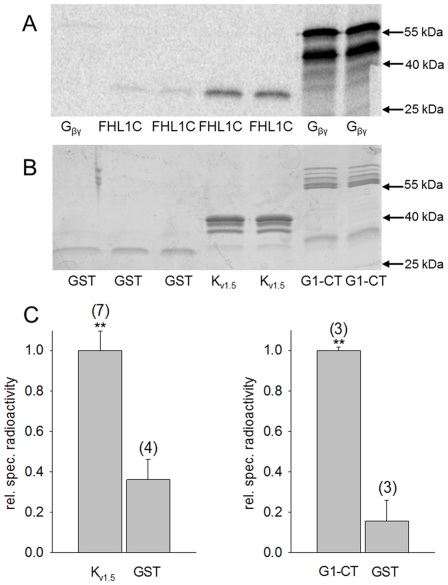
Physical interaction of FHL1C with the K_v1.5_ cytosolic C-terminus. (A) Autoradiography of the dried gel, showing the amount of radioactive protein associated with the bait. As radioactive protein either FHL1C or the ß/γ subunits of heterotrimeric G-protein (G_ß/γ_; control) was used as indicated at the bottom of the lanes. (B) Commassie Brilliant Blue staining of the SDS gel shown in panel A. Bait proteins used are indicated at the bottom of the lanes: GST protein alone (GST); GST-K_v1.5_ C-terminus fusion protein (K_v1.5_); C-terminus of G-protein activated inwardly rectifying potassium channel (GIRK1) fused with GST (G1-CT) as positive control. (C) Statistics of the bound radioactivity normalized to the amount of bait protein (relative specific radioactivity) for FHL1C using K_v1.5_ and GST as a bait (left) and G_ß/γ_ using G1-CT and GST as a bait (right). Values represent the mean values from different experiments (N is given in parenthesis above each bar). Significant difference (**p<0.01) between GST and K_v1.5_ /G1-CT.

Subsequently, it was of interest to investigate whether FHL1C physically interacts with K_v1.5_. As a positive control for protein/protein interaction, the pull-down assay was performed using a GST fusion of the cytosolic C-terminus of GIRK1 (isoform 1 of the G-protein activated inwardly rectifying K^+^ channel; a well known direct G-protein effector) as bait and the G-protein ß_1_/γ_2_ dimer (G_ß/γ_) as binding partner [26]. As a negative control, i.e. no specific protein/protein interaction, pull-down between GST alone and G_ß/γ_ or FHL1C was performed. Although minimal non-specific interaction of FHL1C with GST could be seen, when compared to G_ß/γ_ (Figure 5, lane 2/3 vs. lane 1) considerable physical interaction of FHL1C with the C-terminal portion of K_v1.5_ was clearly detected (Figure 5A/B, lane 4/5).

### Functional effect of FHL1 coexpression on heterologously expressed K_v1.5_ channels

**Figure 6:**
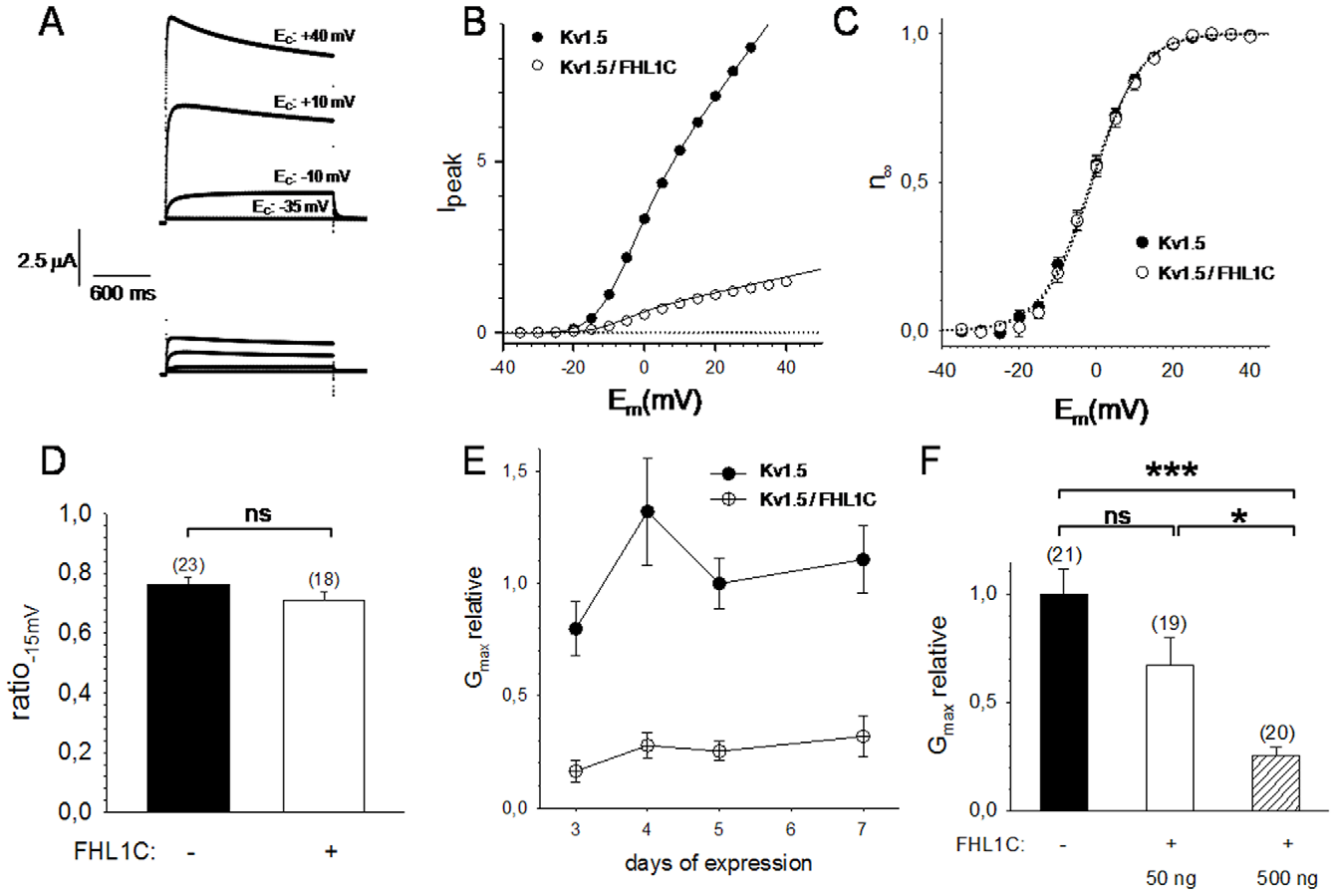
Coexpession of FHL1C reduces currents through K_v1.5_ channels in *Xenopus laevis* oocytes. (A) Representative original current traces recorded from two oocytes expressing either K_v1.5_ alone (upper panel) or K_v1.5_ in combination with FHL1C (lower panel). Each family of current traces originated from four different suprathreshold voltage pulses (−35, −10, +10 and +40 mV). Recordings were taken five days after cRNA injection. (B) I_peak_/E_m_ relation for the oocytes shown in (A). Original data (circles) and fit through the data according to a Boltzmann isotherm (solid lines) are shown. (C) Representative statistics of steady-state activation (n_∞_) five days after cRNA injection (with and w/o 500 ng cRNA encoding FHL1C coexpressed). Circles represent mean values ± SEM from six batches of oocytes (five oocytes per batch and experimental condition) and the dotted lines fits trough the data according to Boltzmann isotherms, respectively. (D) K_v1.5_ inactivation kinetics, same data set as in C. Mean values of the current inactivation ratio (
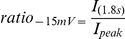
) ± SEM are shown. (E) Time course of the effect of FHL1C coexpression on maximal K^+^ conductance. Data were normalized to G_max_ of oocytes expressing K_v1.5_ alone for five days (mean values ± SEM of six different batches of oocytes are shown); at least five oocytes were recorded from every batch and experimental group. (F) Dose dependent effect of coexpression of FHL1C on G_max_; five days after cRNA injection. The amount of cRNA coinjected, encoding FHL1C, is shown at the bottom. Mean values ± SEM from six different experiments (five oocytes per batch and experimental group) is shown (ns: the difference is statistically not significant, *: p<0.05 and ***: p<0.001, respectively).

In order to test whether FHL1C exerts functional effects on K_v1.5_, both proteins were heterologously coexpressed in the *Xenopus laevis* oocyte system. Functional characterization of K_v1.5_ was performed by assessing its voltage-dependent activation. Most remarkably, coexpression of FHL1C reduced K_v1.5_ currents at all potentials tested, when compared to oocytes expressing K_v1.5_ alone (Figure 6A and B). Such reduction in current magnitude could in principle be brought about by either *(i)* effects of FHL1C association on K_v1.5_ kinetics (i.e. voltage-dependent activation or channel inactivation) or *(ii)* interference of FHL1C with channel biosynthesis and/or trafficking (resulting in reduced insertion of K_v1.5_ complexes into the plasma membrane). Parameters of voltage-dependant activation and channel inactivation are shown in Table 1. Steady-state activation (Figure 6B and C) was analyzed by fitting the measured peak current (I_peak_) as a function of membrane potential (E_m_) to a Boltzmann isotherm resulting in corresponding values for reversal potential (E_rev_), potential for half-maximal activation (E_0.5_), slope of Boltzmann distribution (k) and the conductivity for K^+^ ions at maximal activation (G_max_). Analysis of voltage-dependent activation parameters revealed that neither the potential of half-maximal activation nor the gating charge, i.e. the steepness of the Boltzmann isotherm, was affected by FHL1C (Figure 6C). Also K_v1.5_ inactivation was apparently unchanged upon coexpression with FHL1C (Figure 6D). In conclusion, all analyzed parameters of channel kinetics did not alter when K_v1.5_ was coexpressed with either low or high cRNA levels of FHL1C (Table 1). Interestingly, the maximal conductance (G_max_) of the oocytes for K^+^ ions produced by K_v1.5_ complexes was reduced considerably and statistically significant upon FHL1C coexpression. This reduction was observed during the entire time-span tested after cRNA injections (Figure 6E) and clearly correlated with the amount of coinjected cRNA encoding FHL1C (Figure 6F). Coexpression of oocytes with K_v1.5_ and FHL1C reduced G_max_ by approx. 33% (50 ng FHL1C cRNA/nl) or approx. 75% (500 ng FHL1C cRNA/nl) (Figure 6F).

**Table 1:** Effect of FHL1C coexpression on K_v1.5_ kinetics: reversal potential (E_rev_), potential of half-maximal activation (E_0.5_), slope of Boltzmann isotherm (k) and channel inactivation (ratio _−15 mV_).

	K_v1.5_	K_v1.5_+FHL1C(50 ng cRNA)	K_v1.5_+FHL1C(500 ng cRNA)
E_rev_ (mV)	−33.58±5.71	−36.40±6.25	−36.23±6.20
E_0.5_ (mV)	−1.37±1.94	−1.11±1.53	−1.43±2.34
k (mV^−1^)	6.14±0.12	6.51±0.16	5.95±0.17
ratio _−15 mV_	0.76±0.05	0.74±0.05	0.71±0.04

Values are mean ± SEM of six independent experiments (≥5 oocytes were analysed per experimental day).

Summarizing all data from Figure 6, we conclude that FHL1C coexpression of K_v1.5_ with FHL1C resulted in a reduced membrane insertion of K_v1.5_ complexes into the plasma membrane, without influencing kinetics.

## Discussion

Mutations in FHL1 (previously called skeletal muscle LIM protein 1) cause distinct types of muscular dystrophies e.g. XMPMA [2], which may be associated with cell cycle alterations of myoblasts. We here show a reduced proliferation rate of myoblasts from XMPMA patients; furthermore, an increased number of myoblasts in the G_0_/G_1_ phase is paralleled by low expression of K_v1.5_.

Villalonga and coworkers [14] previously reported that K_v1.5_ is involved in skeletal muscle proliferation. In their studies [14] K_v1.5_ was operative by controlling G_1_ phase progression through a mechanism that involves the CDKIs p21^cip-1^ and p27^kip1^ as well as cyclins A and D_1_. Our study provides additional evidence for a possible involvement of K_v1.5_ in myotube growth; however, the aim of our study was to link myoblast proliferation, expression of K_v1.5_ and a possible interaction with FHL1C, a LIM-domain-containing protein that is abundantly expressed in XMPMA. Although a panel of different interacting proteins as well as putative binding partners have been identified for FHL1 [31], [32], no data are available for FHL1C specifically. We here show for the first time both, a physical and a functional interaction of K_v1.5_ with FHL1C.

A recent paper [33] reported that FHL1 knockdown significantly inhibits proliferation of rat aortic smooth muscle cells. Traffic and subcellular localization regulate ion channel activities [34] and these conditions may affect both skeletal and cardiac muscle tissues [6]–[9]. We here found that FHL1C is localized in the nucleus and the cytoplasm, in the latter colocalizing with K_v1.5_ that can be found mostly in the endoplasmic reticulum and the plasma membrane. Our studies were performed in HL-1 cells, a cellular model that retains important atrial features like contractility and excitability [18]. Our data is the first report on K_v1.5_ expression in myoblasts from patients with a myopathy associated with mutations in FHL1. The presence of intact FHL1C as occurring in XMPMA patients might contribute to the relative mild phenotype when compared to other FHL1 mutation-associated myopathies [2]. We therefore could speculate that FHL1C might compensate either absent or reduced levels of other FHL1 isoforms.

Our *in vitro* data (pull-down assay) confirm a physical interaction of K_v1.5_ with FHL1C. From our observations, we may assume that the two and a half N-terminal LIM domains in FHL1 are responsible for effective interaction with K_v1.5_. Previous genetic analyses of XMPMA patients [6], [9] suggested that FHL1C could play a specific role in cardiomyophathy. In the present study we show that in myoblasts from XMPMA patients (with specific mutations in FHL1) FHL1C is expressed at mRNA levels comparable to controls. However, due to degradation of FHL1A (MUT1) or expression of a truncated FHL1C-like protein (MUT2), FHL1C is likely to increase on a percentage level when compared to total FHL1 expression. Further studies performed in the *Xenopus* oocyte system (Figure 5) demonstrate reduced conductivity of K_v1.5_ (G_max_) indicative for a reduced density of the K^+^ channel within the plasma membrane. This in turn suggests that FHL1C could act as a scaffold protein that modulates K_v1.5_ trafficking to the plasma membrane. We therefore may speculate that increased levels of FHL1C could be the cause for a reduced current in the human atrium leading to enhanced atrial fibrillation in further consequence. K_v1.5_ has been reported to interact with FHL1A [13]. The lack of this FHL1 isoform in XMPMA patients could affect stability of K_v1.5_. Unfortunately, to date, no studies are available on atrial fibrillation and subsequent heart failure in XMPMA patients.

Yang and coworkers [13] have recently reported that coexpression of K_v1.5_ with FHL1A in Chinese hamster ovary cells resulted in increased currents compared to expression of K_v1.5_ alone, findings that are in apparent contrast to our observations coexpressing K_v1.5_ and FHL1C in oocytes. This difference could be due to the fact that we were expressing FHL1C (a protein that lacks two LIM domains in the C-terminal portion) in oocytes where a different processing/trafficking mechanism of FHL1 splice variants may occur. Such alternative processing/trafficking has been reported already for FHL1C but translocation to the nucleus may be mediated by particular protein modifications and/or protein interactions [12], [31]. Another explanation might be the lack of a functional domain, RBP-Jk, in the C-terminal portion of FHL1A (Figure 1). The RBP-Jk moiety, when complexed with the signal-transducing Notch intracellular domain, may act as a transcriptional activator [35], a process recently addressed to occur in myocyte differentiation [36]. Most importantly, KyoT2, the murine homolog of FHL1C, and Notch1 competes with each other for binding to RBP-Jk [30]. Taken these structural elements into account, different effects of the three main FHL1 isoforms on channel activities may not be surprising at all.

In conclusion, we have described the interaction of FHL1C, a specific FHL1 isoform, with K_v1.5_. Since K_v1.5_ expression and functionality are altered in XMPMA patients we assume that FHL1 might regulate channel in both cardiac and skeletal muscle. Absence of FHL1 could result in lack of regulation of the channel and explain at least a part of the (patho)physiology found in XMPMA patients.

## Supporting Information

Figure S1 **Densitometric evaluation of FHL1A and K_v1.5_ expression in human myoblasts.** Immunoreactive bands (shown in Figure 3B) were scanned and their intensity was determined using ImageJ 1.40 g software from Wayne Rasband of the National Institutes of Health (Bethesda, MD, USA). The relative intensity of each band was calculated by dividing the absolute intensity of the respective band by the absolute intensity of the respective loading control band (β-actin). (A) K_v1.5_ expression in control (WT1, WT2) and XMPMA patient (MUT1, MUT2) myoblasts. Values for control samples are set to 1 and for XMPMA patients normalized to corresponding controls. Experiments were performed in triplicates (WT1 and MUT1) and quadruplicates (WT2 and MUT2). (B) FHL1A expression in myoblasts using the same samples as listed in (A). Experiments were performed in triplicates.
(TIF)

Table S1 **Description of primers for the RT-PCR assay, expected amplified fragment size, and annealing temperature.**
(DOC)

Table S2 **Description of primers for DNA constructs, the expected amplified fragment size, and the annealing temperature.**
(DOC)
