# Supplementary figures and images for: Four and a Half LIM Protein 1C (FHL1C): A Binding Partner for Voltage-Gated Potassium Channel Kv1.5
Source: PLoS One. 2011 Oct 28;6(10):e26524. doi: 10.1371/journal.pone.0026524 (PMC3203871; doi:10.1371/journal.pone.0026524)

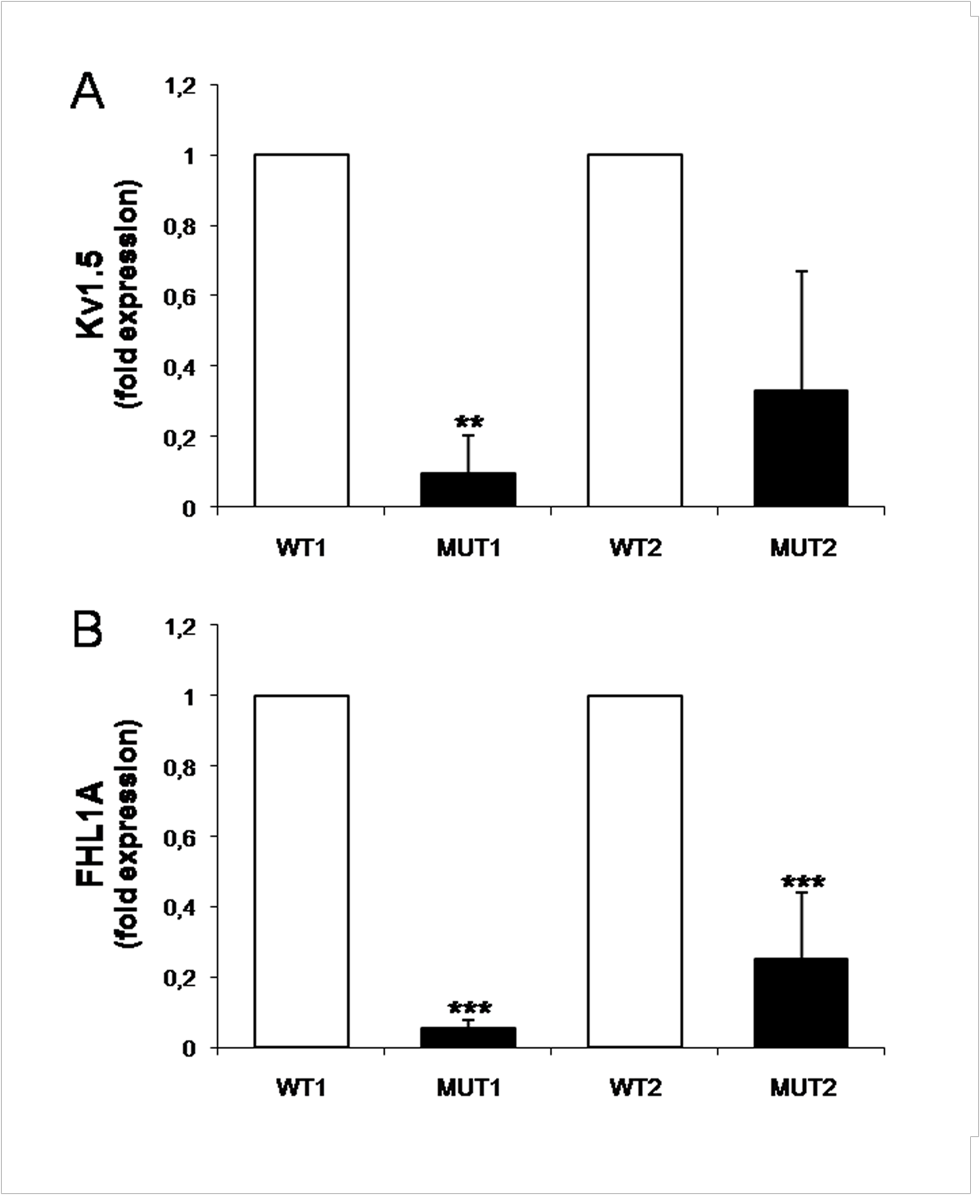

Supplement: Figure S1 — Densitometric evaluation of FHL1A and Kv1.5 expression in human myoblasts. Immunoreactive bands (shown in Figure 3B) were scanned and their intensity was determined using ImageJ 1.40 g software from Wayne Rasband of the National Institutes of Health (Bethesda, MD, USA). The relative intensity of each band was calculated by dividing the absolute intensity of the respective band by the absolute intensity of the respective loading control band (β-actin). (A) Kv1.5 expression in control (WT1, WT2) and XMPMA patient (MUT1, MUT2) myoblasts. Values for control samples are set to 1 and for XMPMA patients normalized to corresponding controls. Experiments were performed in triplicates (WT1 and MUT1) and quadruplicates (WT2 and MUT2). (B) FHL1A expression in myoblasts using the same samples as listed in (A). Experiments were performed in triplicates. (TIF) [file pone.0026524.s001.tif]
