# Supplementary material for: Four and a Half LIM Protein 1C (FHL1C): A Binding Partner for Voltage-Gated Potassium Channel Kv1.5
Source: PLoS One. 2011 Oct 28;6(10):e26524. doi: 10.1371/journal.pone.0026524 (PMC3203871; doi:10.1371/journal.pone.0026524)
Supplement: Table S1 — Description of primers for the RT-PCR assay, expected amplified fragment size, and annealing temperature. (DOC) [file pone.0026524.s002.doc]

| *Gene*  Accession Nr. | Primers | Fragment size (bp) | Annealing temp (°C) |
| --- | --- | --- | --- |
| *FHL1*  NM_001449 | F 5’ GCTGCCTGAAATGCTTTGAC 3’  R 5’GCCAGAAGCGGTTCTTATAGTG 3’ | 105 | 58 |
| *FHL1A*  NM_001449 | F 5’ CTGGATGCAAGAACCCCATC 3’  R 5’ AAAGCGCTTGTTGGCCAG 3’ | 128 | 58 |
| *FHL1C*  NM_001159703 | F 5’ GACTGGAAGCTTCTTCCCTAAAG 3’  R 5’ GCCTTTACCAAACCCTTGTTG 3’ | 105 | 57 |
| *K_v1.5_*  NM_002234 | F 5’ CCGGGAAACGGATCACGAGG 3’  R 5’ GCTGACCTTCCGCTGGACTC 3’ | 100 | 60 |
| *hHPRT1*  NM_000194 | F 5’ CCTGGCGTCGTGATTAGTGAT 3’  R 5’ AGACGTTCAGTCCTGTCCATAA 3’ | 131 | 58 |
