# Supplementary material for: Four and a Half LIM Protein 1C (FHL1C): A Binding Partner for Voltage-Gated Potassium Channel Kv1.5
Source: PLoS One. 2011 Oct 28;6(10):e26524. doi: 10.1371/journal.pone.0026524 (PMC3203871; doi:10.1371/journal.pone.0026524)
Supplement: Table S2 — Description of primers for DNA constructs, the expected amplified fragment size, and the annealing temperature. (DOC) [file pone.0026524.s003.doc]

| *Gene* - vector  Accession Nr. | Primers | Fragment size (bp) | Annealing temp (°C) |
| --- | --- | --- | --- |
| *K_v1.5_* – pBSmxt  NM_002234 | F 5’ TATGAATTCATGGAGATCGCCCTGGTGCC 3’  R 5’ TATGCGGCCGCTCACAAATCTGTTTCCCGGCTGG 3’ | 1840 | 58 |
| *FHL1C*  - pBSmxt  NM_001159703 | F 5’ TATGAATTCATGGCGGAGAAGTTTGACTGCCA 3’  R 5’ TATGCGGCCGCTCACGGAGCATTTTTTGCAGTGGAAG 3’ | 777 | 57 |
| *K_v1.5_* C’ terminus - GST  NM_002234 | F 5’ TATGAATTCAACTACTTCTACCACCGGGAAACG 3’  R 5’ TATGCGGCCGCTCACAAATCTGTTTCCCGGCTGG 3’ | 296 | 57 |
| *K_v1.5_* - pEYFP-N1  NM_002234 | F 5’ TATGAATTCATGGAGATCGC CCTGGTGCC 3’  R 5’ TATGTCGACCAAATCTGTTTCCCGGCTGGTGT 3’ | 1837 | 58 |
| *FHL1C* - pEYFP-N1  NM_001159703 | F 5’ TATGAATTCATGGCGGAGAAGTTTGACTGCCA 3’  R 5’ TATGTCGACCGGAGCATTTTTTGCAGTGGAAGC 3’ | 778 | 57 |
